# Dataset about Southern-Brazilian geopropolis: Physical and chemical perspectives

**DOI:** 10.1016/j.dib.2020.105109

**Published:** 2020-01-09

**Authors:** Bruno Luís Ferreira, Luciano Valdemiro Gonzaga, Luciano Vitali, Gustavo Amadeu Micke, Deise Baggio, Ana Carolina de Oliveira Costa, Roseane Fett

**Affiliations:** a Department of Food Science and Technology, Federal University of Santa Catarina, Florianópolis, SC, Brazil; b Department of Chemistry, Federal University of Santa Catarina, Florianópolis, SC, Brazil

**Keywords:** Stingless bess propolis, *Melipona mondury*, *Melipona quadrifasciata*, *Melipona scutellaris*, *Melipona seminigra*, *Tetragonisca angustula*, Antimicrobial potential, Chemical characterization

## Abstract

The dataset showed in this manuscript belongs to the investigation of the Southern-Brazilian geopropolis of stingless bees. Stingless bees are native species of insects from tropical areas; they produce honey, pollen and geopropolis that is composed of a mix of vegetal extracts, digestive enzymes, and mostly by soil. Used in folk medicine as antiseptic, antioxidant and antimicrobial agent, the composition is due to bee species, climate changes, local flora, and soil type. Moreover, the complex chemical content gives to the geopropolis a bioactive potential, with scavenging characteristics that is important to avoid free radical damages in the human health.

Regarding the importance of exploring new natural matrices sources with bioactive potential, the first approach of chemical characterization of geopropolis is indispensable. Thus, ten samples of Southern-Brazilian geopropolis were analyzed and the bioactive responses obtained were discussed in the accompanying article titled “Southern-Brazilian geopropolis: A potential source of polyphenolic compounds and assessment of mineral composition”. Furthermore, the physicochemical analysis of moisture and ash content, the yield of extraction, the reducing activity and free radical scavenging potential of ethanolic extracts, the antimicrobial activity, and the analysis of HPLC-ESI-MS/MS chromatograms are the main data presented in brief. The data can guide scientists in order to know methods and data for these samples.

**Table:** Specifications Table

Subject	Agricultural and Biological Sciences
Specific subject area	Agricultural and Biological Sciences and Food Science
Type of data	Tables of sampling, moisture and ash content, extract yield, and ethanolic extracts characteristics Figures of antimicrobial activity and HPLC-ESI-MS/MS Chromatograms Tables and figures
How data were acquired	Moisture and ash content: oven SP-400 (SPlabor) and QUIMIS; Extracts yield: analytical scale AB204-S (Mettler Toledo); Reducing activity and free radical scavenging potential: Molecular absorption spectrophotometer UV/Vis (Spectro Vision); HPLC-ESI-MS/MS chromatograms: HPLC model 1200 Series (Agilent Technologies, Alemanha) coupled with mass spectrometer Q Trap 3200 (Applied Biosystems/MDS Sciex, Canada), and Analyst 1.6.2 software; Antimicrobial activity: ultrasonic bath (Unique 1400A);
Data format	Jpg images Analyzed Raw (supplementary material) Raw chromatograms
Parameters for data collection	All geopropolis samples were previously dried in oven 30 °C, 12 h For moisture and ash content were used raw geopropolis samples For reducing activity and free radical scavenging potential, extract yield, HPLC-ESI-MS/MS chromatograms and antimicrobial activity a solid-liquid extraction procedure was used
Description of data collection	The data was collected by measuring the absorbance (UV/Vis) The chromatograms were collected by using Analyst software in the HPLC-ESI-MS/MS system
Data source location	Federal University of Santa Catarina Laboratory of Food Chemistry, Florianópolis, Santa Catarina, Brazil The samples were collected during 2016–2017 at Santa Catarina state (further details below)
Data accessibility	Within the article and in supplementary material
Related research article	Ferreira B. L., Gonzaga L. V., Vitali L., Micke G. A., Maltez H. F., Ressureição C., Costa A. C. O., Fett R. Southern-Brazilian geopropolis: A potential source of polyphenolic compounds and assessment of mineral composition Food Research International 10.1016/j.foodres.2019.108683

**Table:** 

**Value of the Data** • Data about geopropolis from stingless bees are appropriate regarding the lack of scientific information about this natural product. Also, taking into account the bioactive potential of slight unexplored natural sources. • The data can serve as an indication for further applications of geopropolis in food matrices or even for pharmaceutical purposes, especially regarding the profile of polyphenolic analysis through the chromatograms and the methods used to access these profiles. • For further experiments using these data as insight, scientists can recognize the value of geopropolis samples and develop new set of experiments using other bee species worldwide, extending the methods showed forward. Besides, a comparison of the reference data with different sources. • The sample preparation of methods below is useful in order to access each geopropolis characteristic. • Each experiment in this brief was carefully performed in order to keep the accuracy of data, minimizing negative or positive assumptions.

## 1 Data

The dataset in this article describes some physical and chemical characteristics of ten samples of geopropolis. Table 1 describes the sample collection with general information about bee species, the geographical location where the geopropolis were collected, also the code used to refer to each sample. The percentage of moisture and ash content are showed in Table 2. In Table 3, it is describing the yield of extraction regarding two different solvents and three different periods for each geopropolis sample, elsewhere the statistical standard deviation and analysis of means by Tukey's test (95%). Regarding the ethanol as solvent, Table 4 brings the reducing activity and the free radical scavenging potential of geopropolis samples in three different periods of extraction. The mass/charge relation of each polyphenolic compound indicating the parent íon and the quantification íon, in addition to the retention time is in the Table 5.

**Table 1:** Samples of geopropolis, location, and reference codes.

Stingless Bee Species	Code	Location	Latitude	Longitude	Altitude
*Melipona mondury*	MMS	Santa Rosa de Lima (C)	28°02′21″ south	49°7′40″ west	240 m
	MMI	Iporã do Oeste (A)	26°8′8″ south	53°53′5″ west	557 m
*Melipona quadrifasciata*	MQS	Santa Rosa de Lima (C)	28°02′21″ south	49°7′40″ west	240 m
	MQR	Rio do Sul (B)	27°12′51″ south	49°38′35″ west	340 m
	MQF	Florianópolis (D)	27°35′48″ south	48°32′57″ west	0 m
	MQI	Iporã do Oeste (A)	26°8′8″ south	53°53′5″ west	557 m
*Melipona scutellaris*	MSS	Santa Rosa de Lima (C)	28°02′21″ south	49°7′40″ west	240 m
	MSI	Iporã do Oeste (A)	26°8′8″ south	53°53′5″ west	557 m
*Melipona seminigra*	MSeS	Santa Rosa de Lima (C)	28°02′21″ south	49°7′40″ west	240 m
*Tetragonisca angustula*	TAI	Iporã do Oeste (A)	26°8′8″ south	53°53′5″ west	557 m

**Table 2:** Moisture and ash content of crude geopropolis samples.

Samples	Moisture (%)	Ash content (%)
MMS	3.51 ± 0.10	80.18 ± 2.50
MMI	2.60 ± 0.02	77.51 ± 0.73
MQS	3.79 ± 0.28	66.01 ± 0.58
MQR	4.77 ± 0.06	51.97 ± 0.76
MQF	3.65 ± 0.17	65.96 ± 0.68
MQI	3.32 ± 0.10	58.32 ± 0.38
MSS	3.23 ± 0.26	78.29 ± 0.30
MSI	3.21 ± 0.02	70.17 ± 1.23
MSeS	8.80 ± 0.19	71.48 ± 0.84
TAI	4.12 ± 0.11	2.23 ± 0.01

Data showed by percentage (mean ± standard deviation, n = 3). Raw data available in supplementary material 1.

**Table 3:** Yield of extraction of geopropolis samples regarding pure ethanol and pure methanol as solvents over storage time.

Samples	Ethanolic extraction			Methanolic extraction		
	10 days	20 days	30 days	10 days	20 days	30 days
MMS	10.91 ± 1.71^a^	11.31 ± 1.14^a^	9.70 ± 0.01^a^	12.47 ± 0.01^a^	14.96 ± 0.78^b^	12.05 ± 0.59^a^
MMI	64.77 ± 6.33^a^	42.68 ± 3.48^a^	29.96 ± 0.58^a^	40.84 ± 2.31^a^	46.56 ± 5.78^a^	47.38 ± 4.62^a^
MQS	179.52 ± 1.15^a^	174.65 ± 5.64^a^	173.02 ± 1.15^a^	157.96 ± 0.01^a^	158.79 ± 3.53^a^	165.44 ± 8.23^a^
MQR	338.83 ± 1.76^a^	336.75 ± 7.06^a^	350.05 ± 14.11^a^	331.17 ± 0.58^a^	329.54 ± 5.20^a^	355.67 ± 13.28^a^
MQF	229.38 ± 0.58^a^	231.85 ± 0.58^b^	233.91 ± 0.01^c^	237.92 ± 4.10^a^	239.57 ± 1.76^a^	261.50 ± 2.34^b^
MQI	185.80 ± 3.49^a^	190.73 ± 11.63^a^	203.48 ± 12.21^a^	192.74 ± 2.32^a^	212.01 ± 0.58^b^	220.22 ± 1.74^c^
MSS	29.73 ± 1.14^a^	26.11 ± 0.57^a^	25.31 ± 0.57^a^	25.59 ± 1.17^a^	36.32 ± 0.01^b^	23.11 ± 0.01^a^
MSI	59.48 ± 1.15^a^	67.22 ± 7.49^a^	64.77 ± 6.34^a^	114.97 ± 1.76^a^	136.97 ± 9.39^a^	117.46 ± 0.59^a^
MSeS	23.17 ± 0.01^a^	21.32 ± 1.12^a^	22.90 ± 1.12^a^	15.78 ± 1.17^a^	25.09 ± 0.23^b^	10.80 ± 1.17^c^
TAI	395.95 ± 8.78^a^	398.43 ± 1.76^a^	403.40 ± 1.76^a^	196.37 ± 2.92^a^	213.32 ± 0.01^a^	202.16 ± 6.43^a^

Data showed as mg g^-1^ 89 (mean±standard deviation, n=3). Different letters in the same line regarding the same solvent indicate statistical difference according to Tukey's test (95%). Raw data available in supplementary material 2.

**Table 4:** Reducing activity and the free radical scavenging potential of crude geopropolis samples in three different periods of extraction using ethanol as extractor agent.

Samples	Days of extraction	Ethanolic extraction		
		Reducing activity (GAE mg 100g^−1^)	Free radical scavenging potential	
			AAE mg 100g^−1^	TE mg 100g^−1^
MMS	10	62.68 ± 0.77	75.83 ± 1.15	111.32 ± 1.68
	20	65.40 ± 1.71	74.26 ± 2.83	109.01 ± 4.16
	30	65.79 ± 2.97	77.96 ± 0.44	114.44 ± 0.64
MMI	10	479.15 ± 31.05	549.86 ± 10.41	806.95 ± 15.29
	20	491.73 ± 2.90	528.79 ± 7.89	775.99 ± 11.59
	30	549.81 ± 13.31	602.88 ± 7.37	884.84 ± 10.83
MQS	10	1023.37 ± 15.37	1164.56 ± 3.84	1709.16 ± 5.64
	20	1021.44 ± 39.22	1149.70 ± 9.03	1687.31 ± 13.26
	30	1046.61 ± 8.71	1218.26 ± 13.30	1788.04 ± 19.53
MQR	10	1067.96 ± 134.74	254.11 ± 3.42	372.88 ± 5.03
	20	1258.21 ± 26.53	286.24 ± 1.32	420.08 ± 1.94
	30	1319.99 ± 4.49	325.15 ± 1.00	477.24 ± 1.46
MQF	10	1651.45 ± 44.52	3057.62 ± 32.88	4487.67 ± 48.31
	20	1748.59 ± 33.65	3390.98 ± 26.96	4977.41 ± 39.61
	30	2069.16 ± 261.20	3613.46 ± 22.16	5304.25 ± 32.56
MQI	10	1555.32 ± 47.62	1624.30 ± 24.74	2382.00 ± 36.34
	20	1508.78 ± 26.66	1665.46 ± 17.76	2442.45 ± 26.09
	30	1557.26 ± 42.88	1954.49 ± 6.93	2867.08 ± 10.18
MSS	10	439.05 ± 10.60	977.36 ± 6.50	1434.12 ± 9.56
	20	456.69 ± 23.34	1018.49 ± 19.83	1494.53 ± 29.13
	30	460.61 ± 30.17	1111.43 ± 4.11	1631.07 ± 6.04
MSI	10	1652.80 ± 24.00	1927.42 ± 38.60	2827.34 ± 56.70
	20	1395.28 ± 17.61	1780.25 ± 8.49	2611.13 ± 12.47
	30	1326.09 ± 53.26	2065.76 ± 11.47	3030.58 ± 16.86
MSeS	10	67.83 ± 1.60	63.05 ± 1.58	92.54 ± 2.32
	20	67.73 ± 0.60	64.96 ± 0.95	95.34 ± 1.40
	30	71.09 ± 4.58	70.56 ± 1.70	103.58 ± 2.50
TAI	10	1301.95 ± 109.71	347.71 ± 9.84	510.39 ± 14.46
	20	1231.68 ± 106.86	315.44 ± 12.76	462.99 ± 18.74
	30	1370.27 ± 129.01	349.03 ± 20.63	512.34 ± 30.31

Data showed as mg 100 g^−1^ (mean ± standard deviation, n = 3). Raw data available in supplementary material 3.

**Table 5:** Mass/charge relation of each polyphenolic compound analized in the geopropolis samples.

Polyphenolic compound	Parent íon (*m/z*) - Q1	Quantitative íon (*m/z*) - Q3	Retention time (min)
Gallic ac	168.908	125	3.98
Protocatechuic ac	152.921	109	6.95
Mandelic ac	150.996	107	7.86
Catechin	289.045	109	8.82
4-(Hydroxymethyl)benzoic ac	150.967	107	8.84
Chlorogenic ac	353.155	191	9.19
Epicatechin	288.954	109	9.41
Caffeic ac	178.927	135	9.45
Vanillic ac	166.923	108	9.65
Syringic ac	196.939	121.1	10.01
Epicatechin gallate	441.6	168.9	10.15
Fustin	286.969	109	10.32
Vanilin	150.958	136	10.42
p-Coumaric ac	162.926	119.1	10.46
4-aminobenzoic ac	135.995	91.9	10.47
α-Methoxyphenylacetic ac	164.976	121.1	10.51
Taxifolin	303.019	125.1	10.7
Rutin	609.242	300.1	10.72
Ferulic ac	192.957	134	10.73
Syringaldehyde	180.94	151	10.76
Umbelliferone	160.941	133.1	10.78
Rosmarinic ac	359.082	161	10.83
Isoquercitrin	463.155	300	10.83
Quercetin	300.968	151	10.84
Sinapic ac	223.011	148.8	10.87
Salicylic ac	136.942	93	10.99
Escopoletin	190.972	176	10.99
Resveratrol	226.999	142.9	11.14
Naringin	580.276	151	11.18
Miricetrin	316.995	151	11.24
Aromadendrin	287.004	125	11.29
Coniferaldehyde	177.015	162	11.31
p-Anisic ac	150.947	136	11.34
Sinapaldehyde	207.04	177	11.39
Ellagic ac	300.959	145	11.71
Cinnamic ac	146.952	102.9	11.8
Eriodictyol	186.97	151	11.85
Kaempferol	284.995	93	12.34
Naringenin	270.985	151.1	12.37
Apigenin	268.992	117.1	12.62
Hispidulin	298.957	284	12.72
Galangin	268.981	117	13.44
Pinocembrin	255.051	65	13.59
Chrysin	252.988	62.9	13.88
Carnosol	329.167	285.2	14.32

**Fig. 1:**
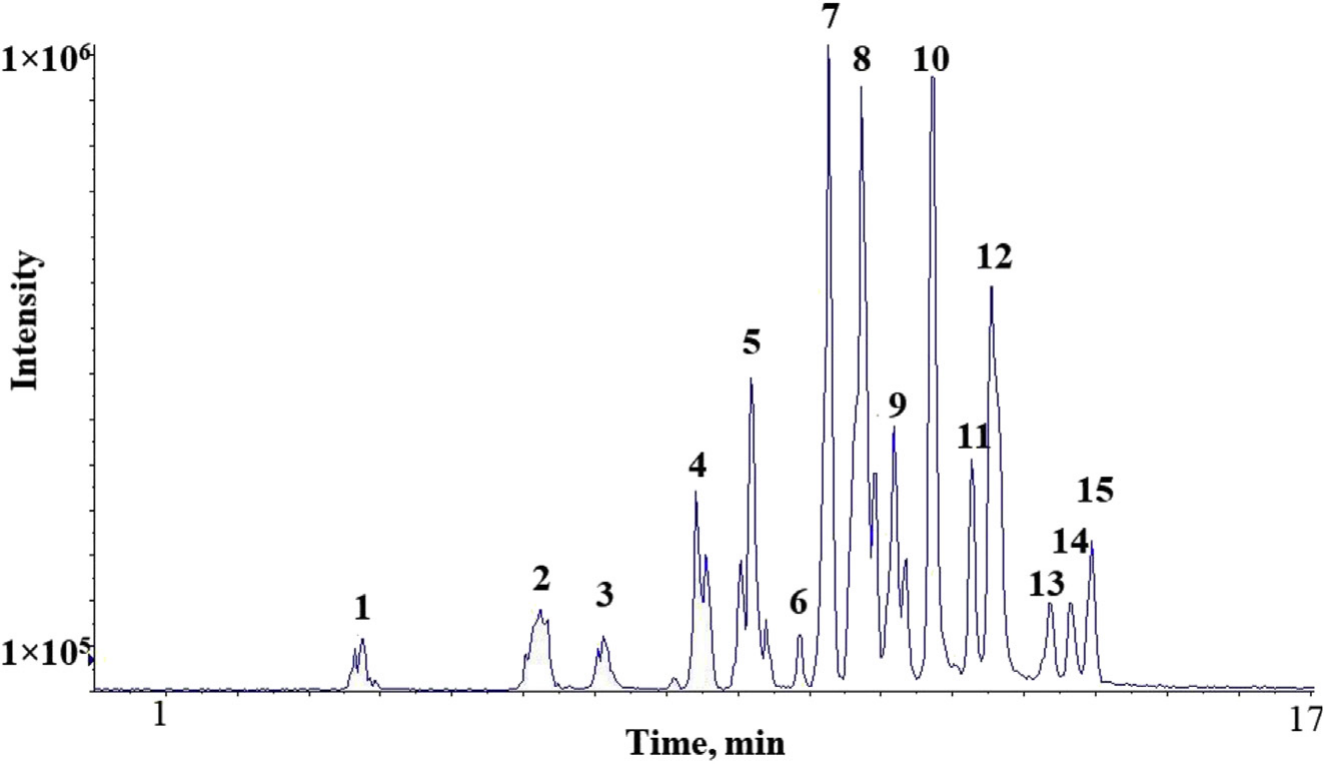
HPLC-ESI-MS/MS chromatograms of polyphenolic standards. *Polyphenolic compounds on a mix solution of standards: (1) Gallic ac. (2) Protocatechuic ac. (3) Mandelic ac. (4) Catechin, 4-(Hydroxymethyl)benzoic ac. (5) Vanillic ac, Caffeic ac, Chlorogenic ac, Epicatechin. (6) Syringic ac. (7) Vanilin, 4-aminobenzoic ac, p-Coumaric ac, α-Methoxyphenylacetic ac, Syringaldehyde, Taxifolin, Epicatechin gallate, Rutin. (8) Salicylic ac, Umbelliferone, Escopoletin, Ferulic ac, Sinapic ac, Rosmarinic ac, Isoquercitrin, Naringin, Fustin. (9) Aromadendrin, p-Anisic ac, Coniferaldehyde, Sinapaldehyde, Resveratrol, Miricetrin. (10) Cinnamic ac, Eriodictyol, Ellagic ac, Quercetin. (11) Galangin, Naringenin, Kaempferol. (12) Apigenin, Hispidulin. (13) Pinocembrin. (14) Chrysin. (15) Carnosol. The chemical structure of each polyphenolic compound are in the supplementary material of [4].

**Fig. 2:**
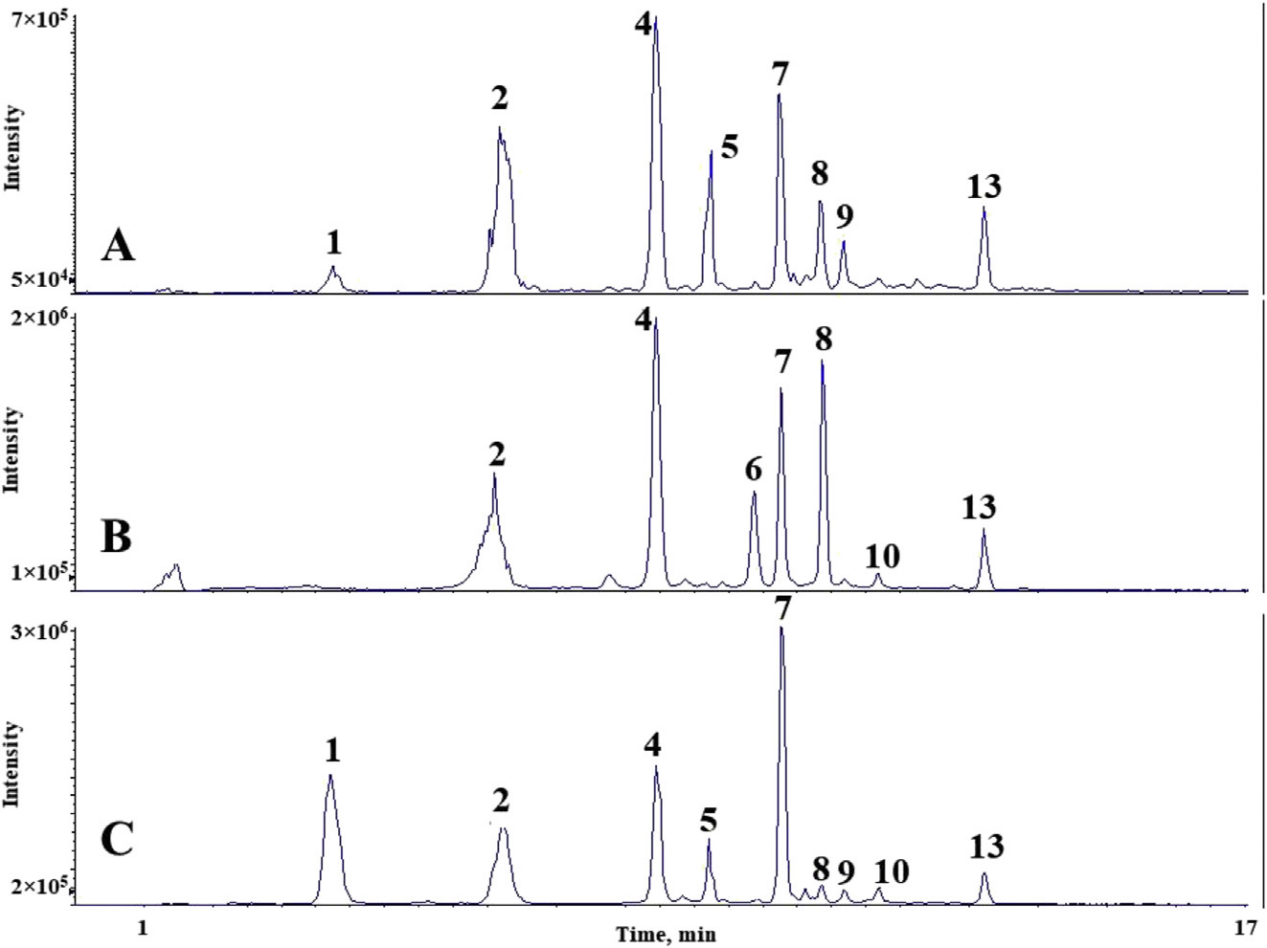
Polyphenolic profile of MMS sample (HPLC-ESI-MS/MS chromatogram). (A) Free polyphenolic profile. (B) Polyphenolic profile after acid hydrolysis. (C) Polyphenolic profile after alkaline hydrolysis. Each peak can possess more than one polyphenolic compound, according to Fig. 1. The results regarding the polyphenolic quantification are in the original paper [4].

**Fig. 3:**
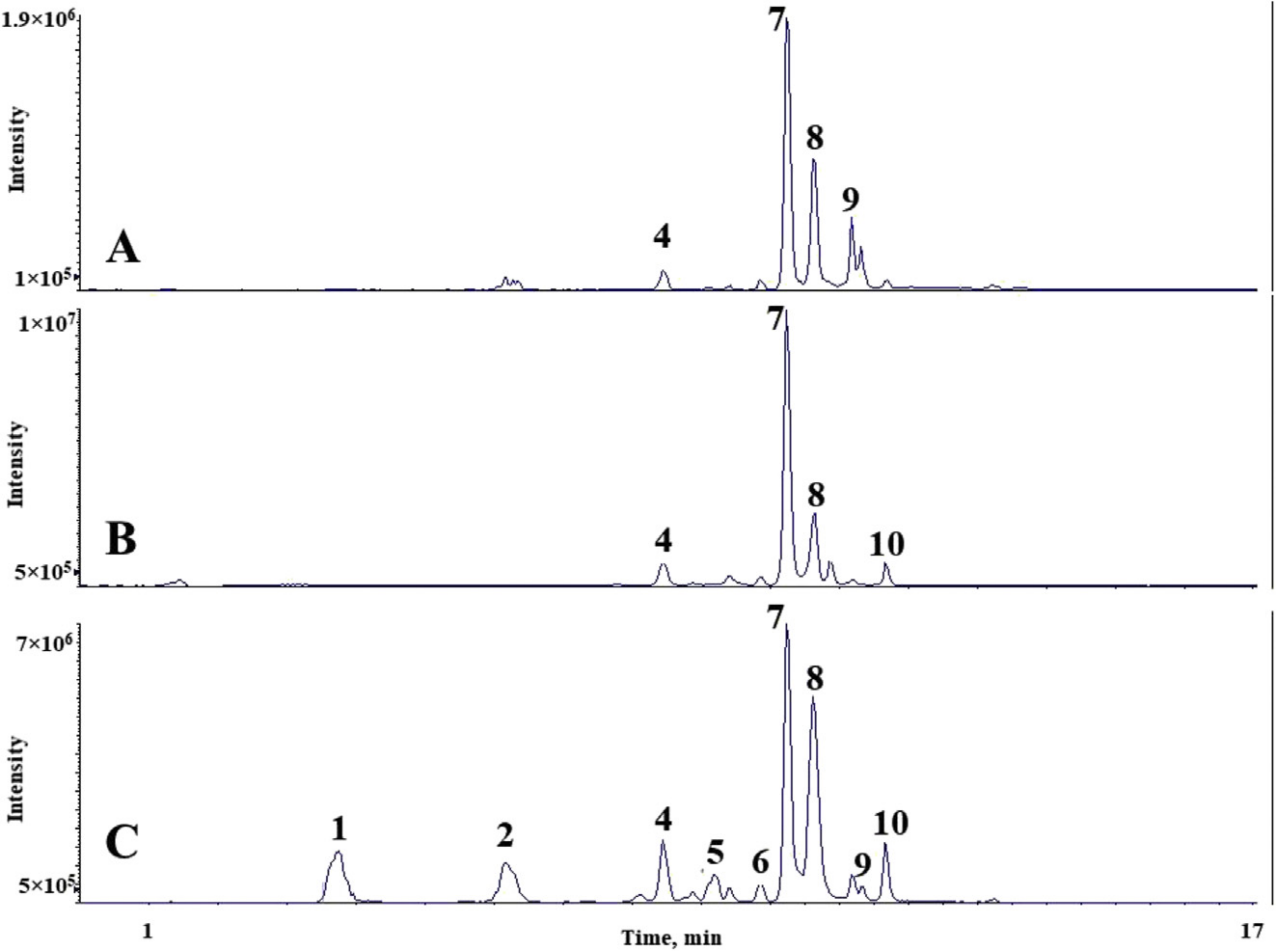
Polyphenolic profile of MMI sample (HPLC-ESI-MS/MS chromatogram). (A) Free polyphenolic profile. (B) Polyphenolic profile after acid hydrolysis. (C) Polyphenolic profile after alkaline hydrolysis. Each peak can possess more than one polyphenolic compound, according to Fig. 1. The results regarding the polyphenolic quantification are in the original paper [4].

**Fig. 4:**
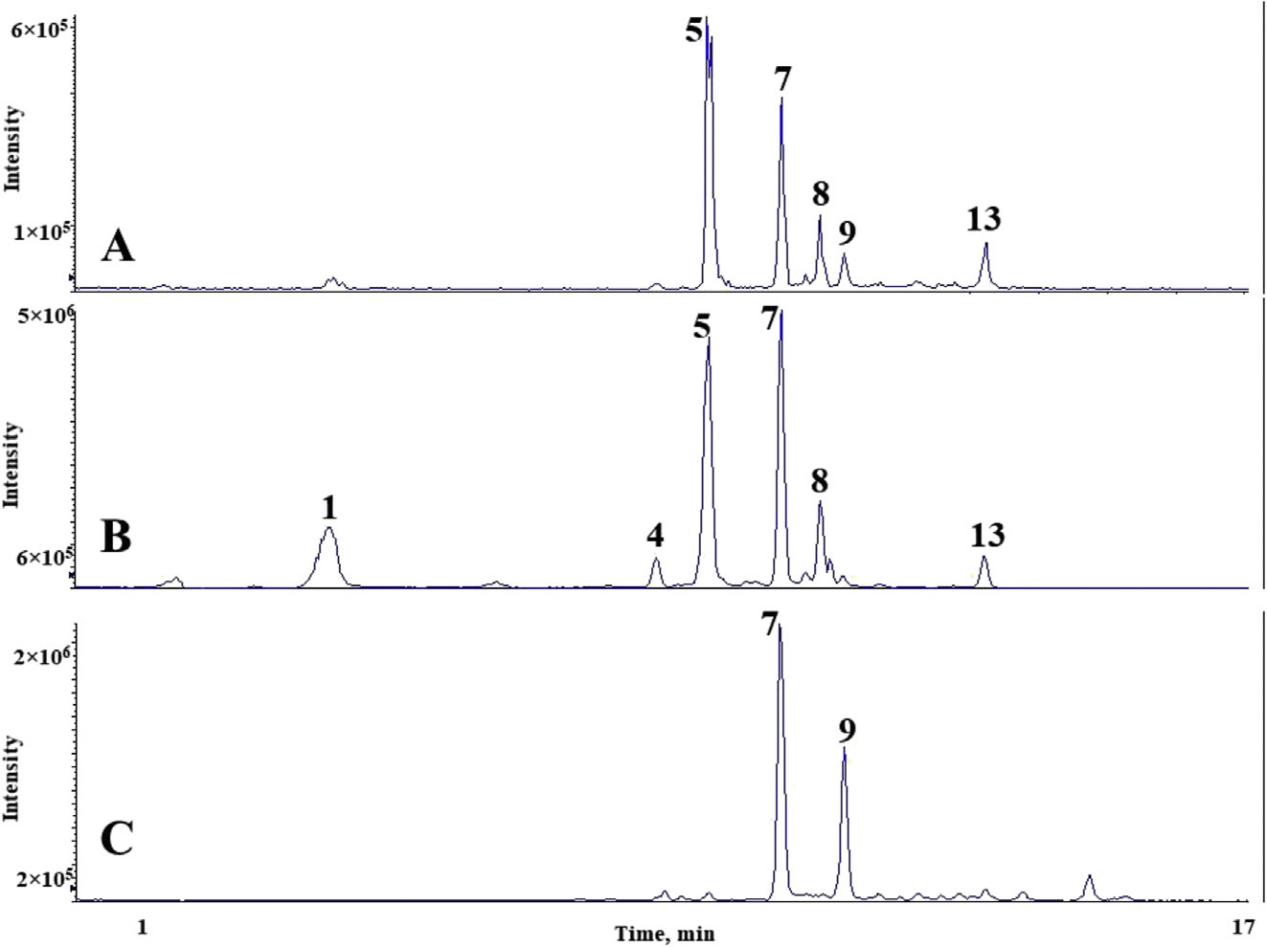
Polyphenolic profile of MQS sample (HPLC-ESI-MS/MS chromatogram). (A) Free polyphenolic profile. (B) Polyphenolic profile after acid hydrolysis. (C) Polyphenolic profile after alkaline hydrolysis. Each peak can possess more than one polyphenolic compound, according to Fig. 1. The results regarding the polyphenolic quantification are in the original paper [4].

**Fig. 5:**
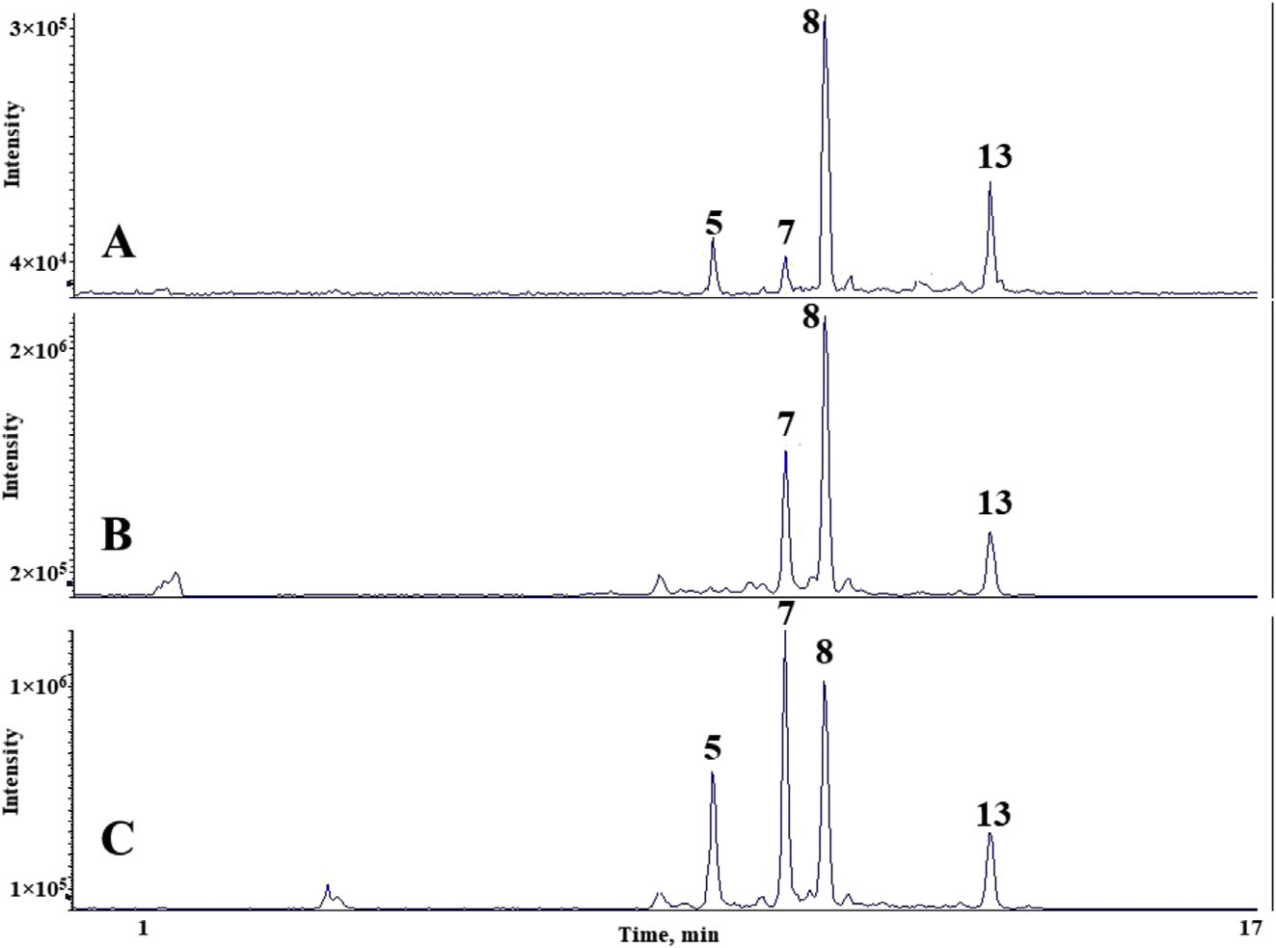
Polyphenolic profile of MQR sample (HPLC-ESI-MS/MS chromatogram). (A) Free polyphenolic profile. (B) Polyphenolic profile after acid hydrolysis. (C) Polyphenolic profile after alkaline hydrolysis. Each peak can possess more than one polyphenolic compound, according to Fig. 1. The results regarding the polyphenolic quantification are in the original paper [4].

**Fig. 6:**
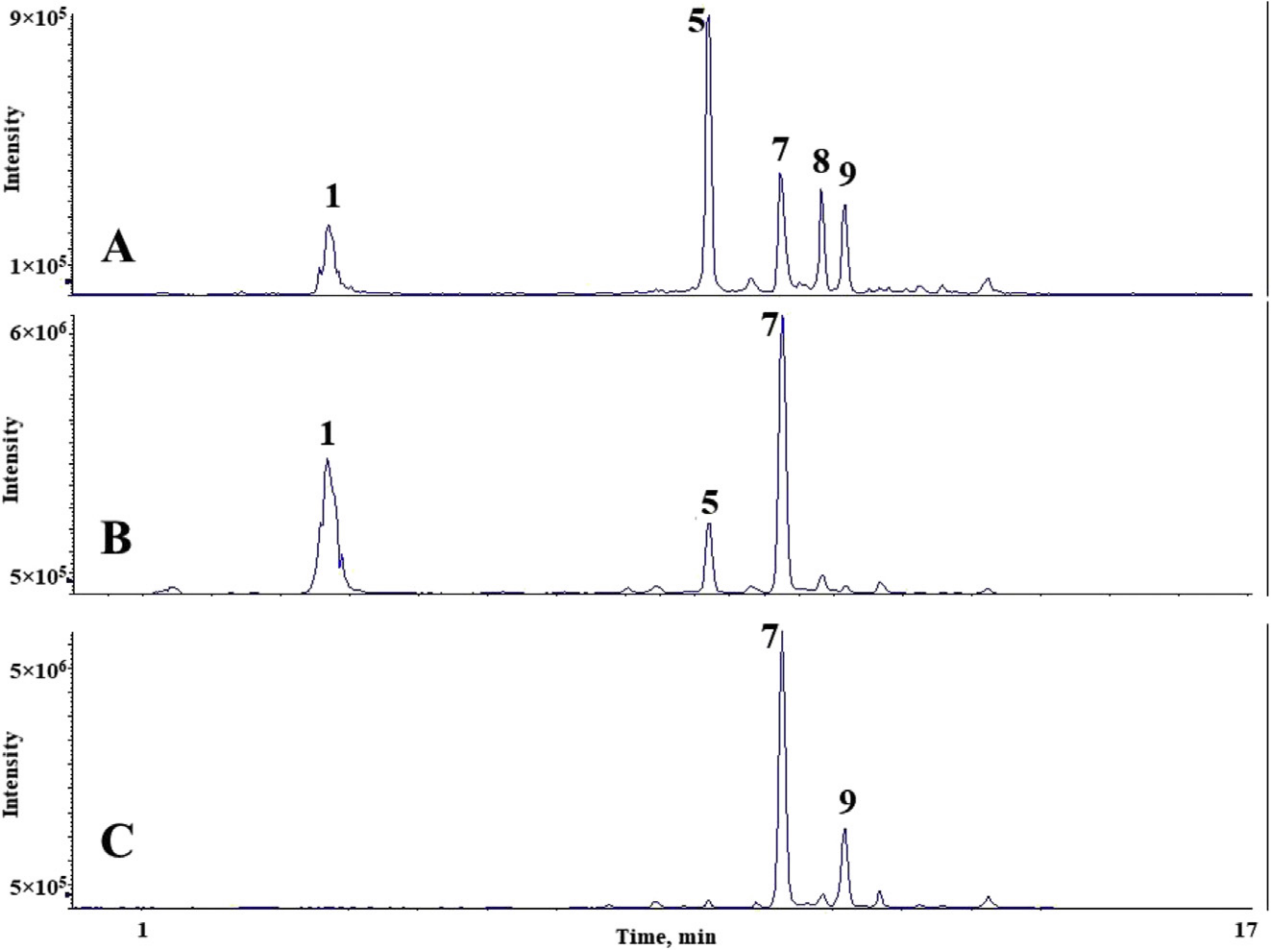
Polyphenolic profile of MQF sample (HPLC-ESI-MS/MS chromatogram). (A) Free polyphenolic profile. (B) Polyphenolic profile after acid hydrolysis. (C) Polyphenolic profile after alkaline hydrolysis. Each peak can possess more than one polyphenolic compound, according to Fig. 1. The results regarding the polyphenolic quantification are in the original paper [4].

**Fig. 7:**
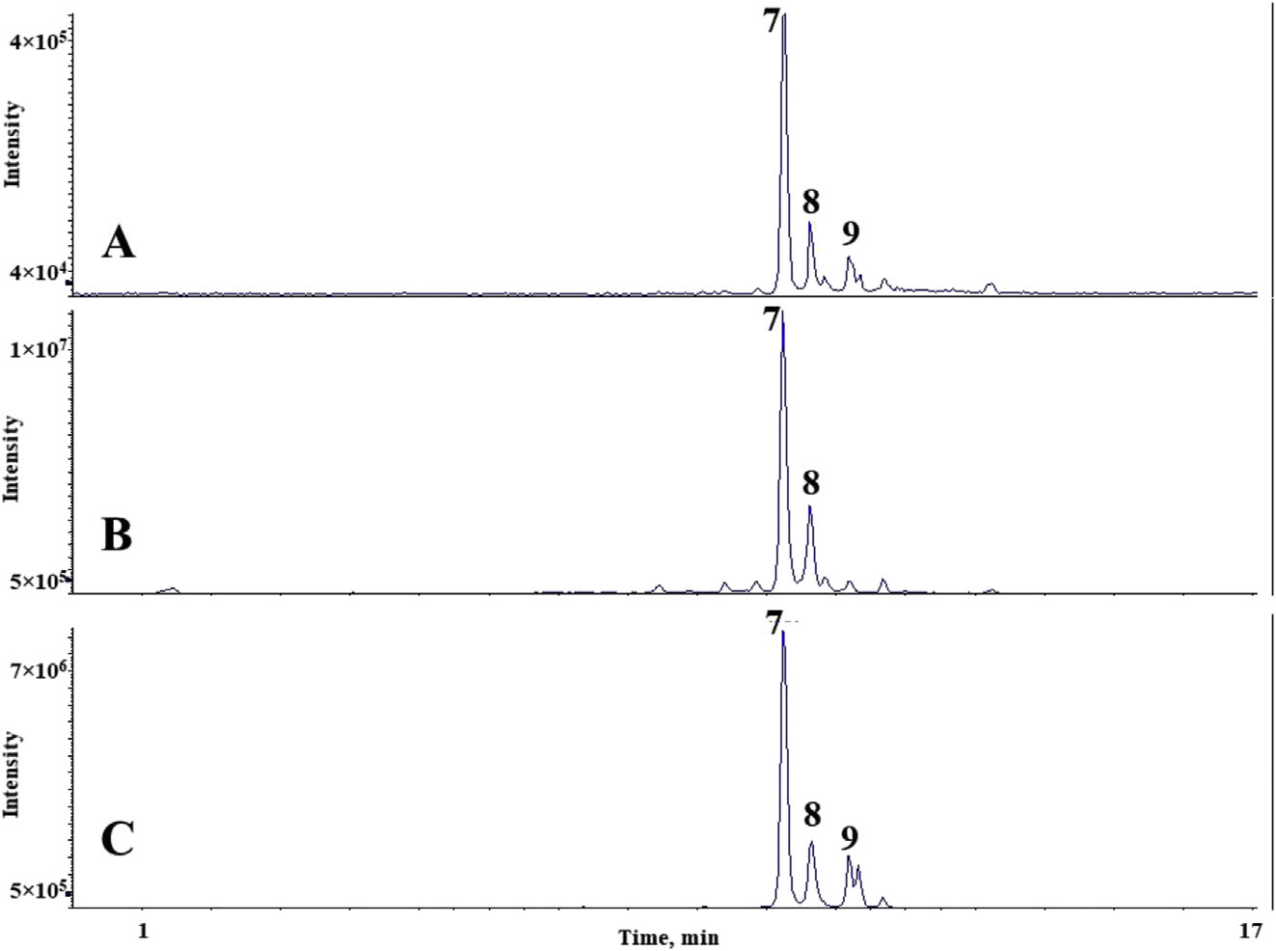
Polyphenolic profile of MQI sample (HPLC-ESI-MS/MS chromatogram) (A) Free polyphenolic profile. (B) Polyphenolic profile after acid hydrolysis. (C) Polyphenolic profile after alkaline hydrolysis. Each peak can possess more than one polyphenolic compound, according to Fig. 1. The results regarding the polyphenolic quantification are in the original paper [4].

**Fig. 8:**
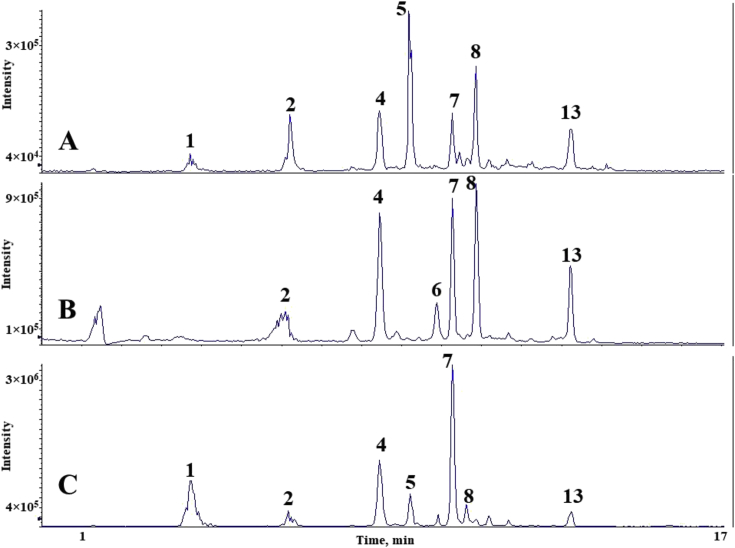
Polyphenolic profile of MSS sample (HPLC-ESI-MS/MS chromatogram). (A) Free polyphenolic profile. (B) Polyphenolic profile after acid hydrolysis. (C) Polyphenolic profile after alkaline hydrolysis. Each peak can possess more than one polyphenolic compound, according to Fig. 1. The results regarding the polyphenolic quantification are in the original paper [4].

**Fig. 9:**
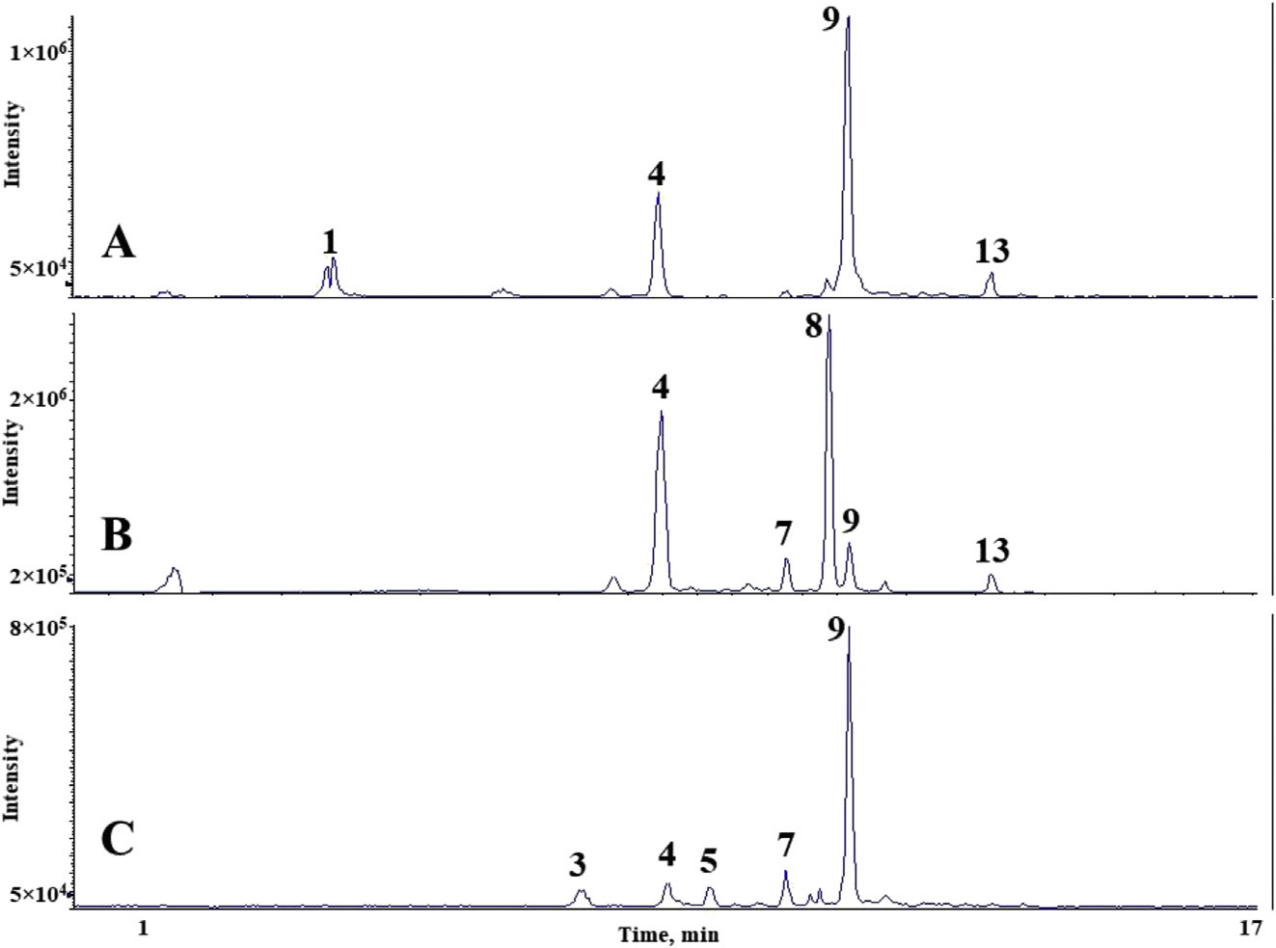
Polyphenolic profile of MSI sample (HPLC-ESI-MS/MS chromatogram). (A) Free polyphenolic profile. (B) Polyphenolic profile after acid hydrolysis. (C) Polyphenolic profile after alkaline hydrolysis. Each peak can possess more than one polyphenolic compound, according to Fig. 1. The results regarding the polyphenolic quantification are in the original paper [4].

**Fig. 10:**
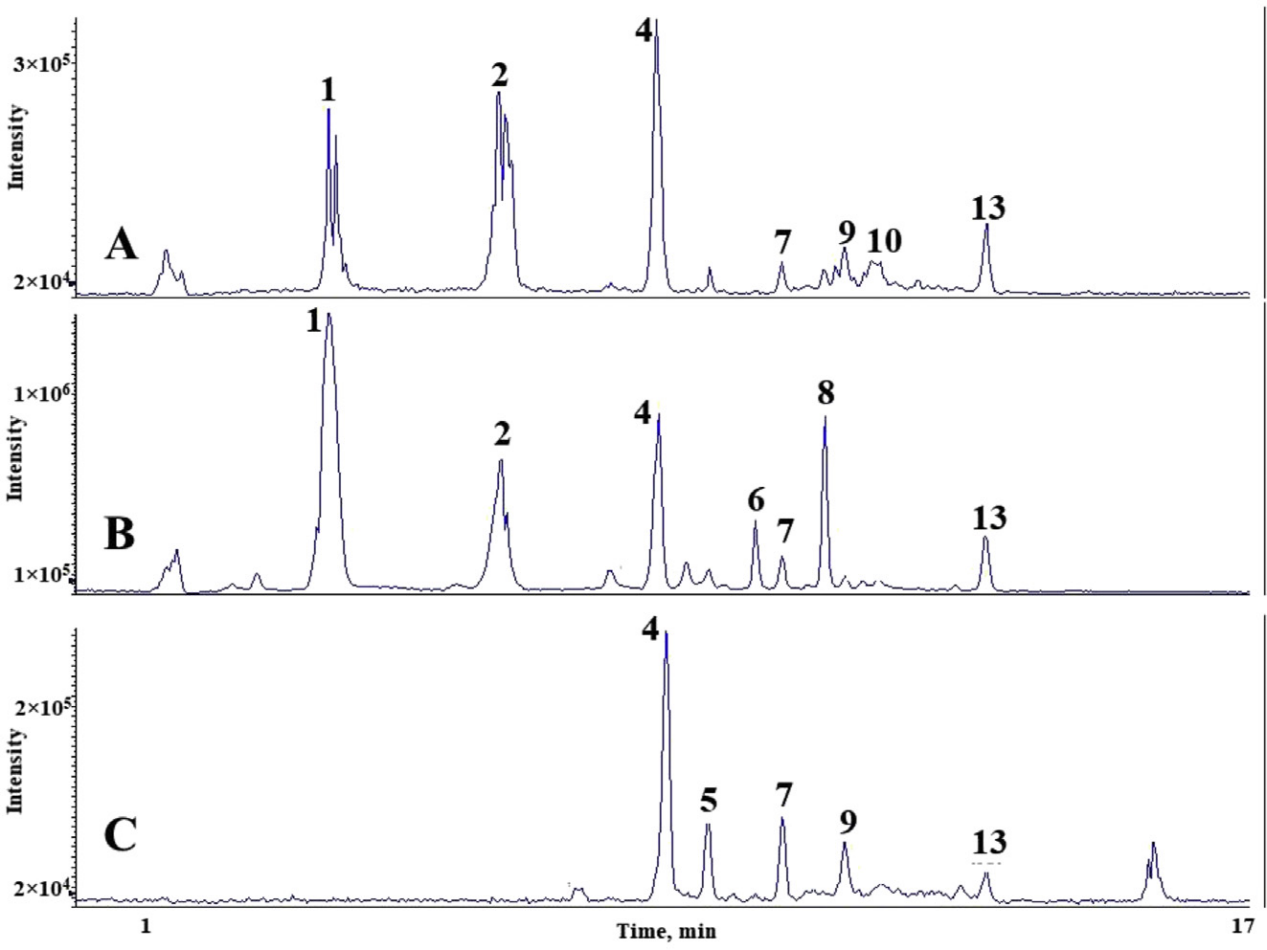
Polyphenolic profile of MSeS sample (HPLC-ESI-MS/MS chromatogram). (A) Free polyphenolic profile. (B) Polyphenolic profile after acid hydrolysis. (C) Polyphenolic profile after alkaline hydrolysis. Each peak can possess more than one polyphenolic compound, according to Fig. 1. The results regarding the polyphenolic quantification are in the original paper [4].

**Fig. 11:**
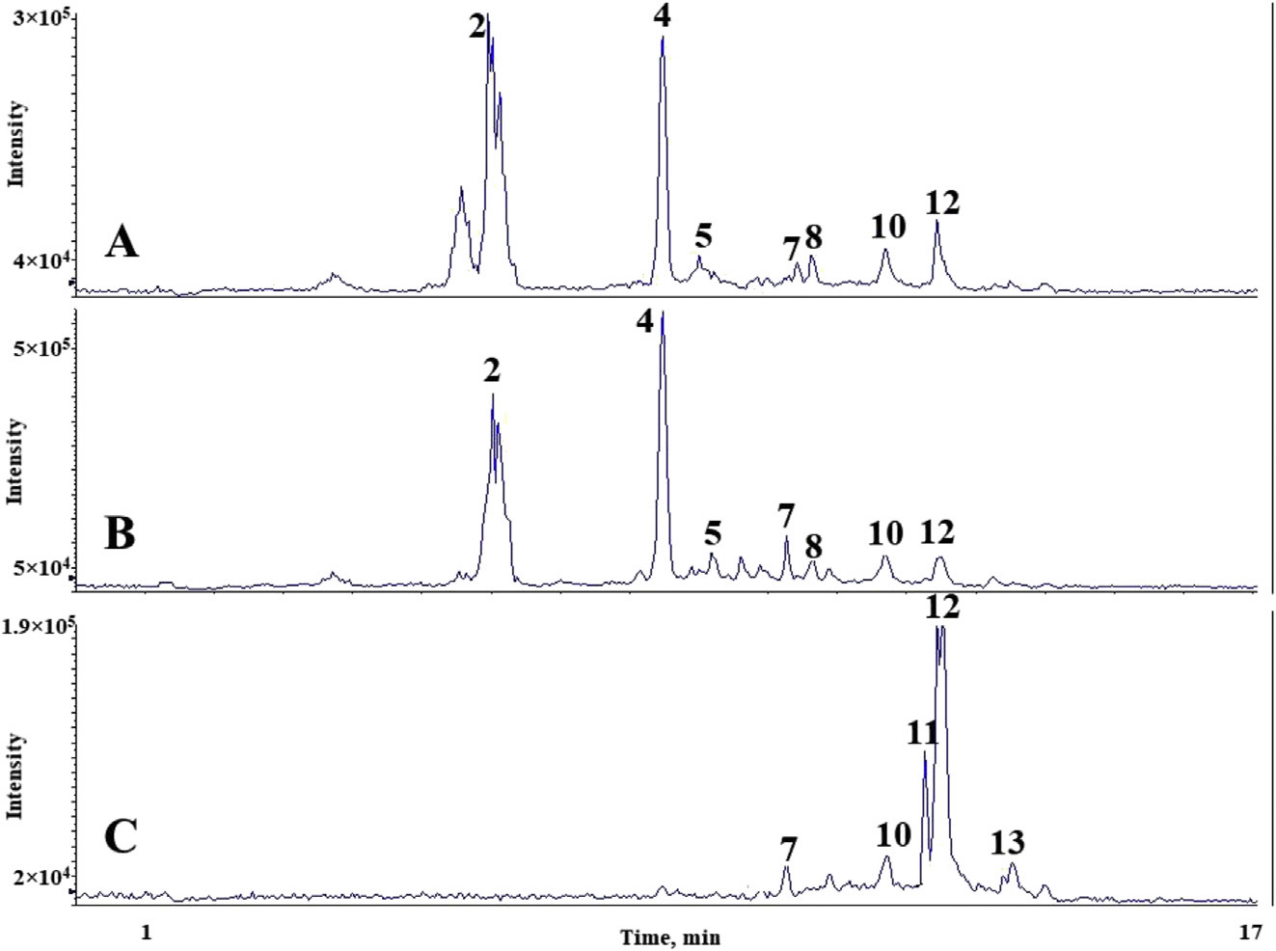
Polyphenolic profile of TAI sample (HPLC-ESI-MS/MS chromatogram). (A) Free polyphenolic profile. (B) Polyphenolic profile after acid hydrolysis. (C) Polyphenolic profile after alkaline hydrolysis. Each peak can possess more than one polyphenolic compound, according to Fig. 1. The results regarding the polyphenolic quantification are in the original paper [4].

**Fig. 12:**
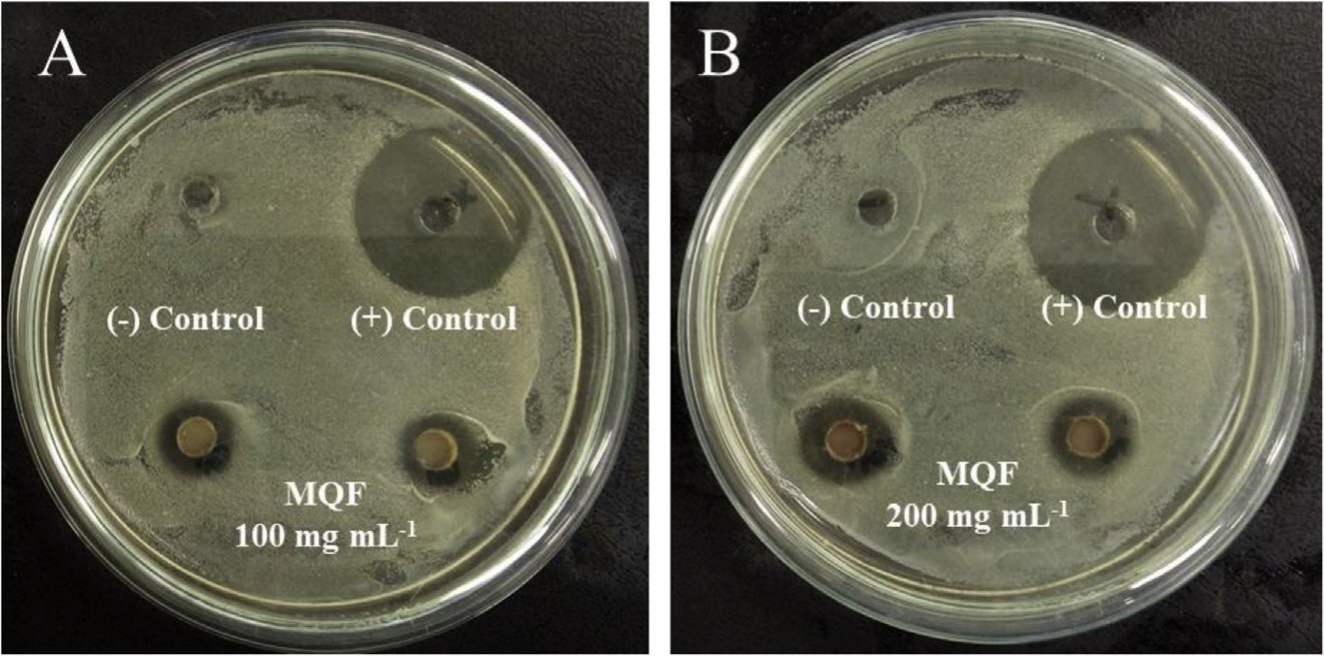
Antimicrobial potential of MQF sample. Sample MQF (100 (A) and 200 mg mL-1 (B)) showed inhibition halo formation surrounding the well containing *S. aureus*.

The chromatograms of polyphenolic analysis are in Fig. 1, Fig. 2, Fig. 3, Fig. 4, Fig. 5, Fig. 6, Fig. 7, Fig. 8, Fig. 9, Fig. 10, Fig. 11. The analytical standards separation is represented in Fig. 1. Fig. 2, Fig. 3, Fig. 4, Fig. 5, Fig. 6, Fig. 7, Fig. 8, Fig. 9, Fig. 10, Fig. 11 are the geopropolis samples, regarding the use of three different strategies to access the polyphenolic composition of each: free polyphenolics, and bonded polyphenolics by using acid and alkaline hydrolysis. Finally, Fig. 12 showed the antimicrobial potential of geopropolis samples.

## 2 Experimental design, materials, and methods

Initially 10 geopropolis samples of *Melipona mondury* (n = 2), *Melipona quadrifasciata* (n = 4), *Melipona scutellaris* (n = 2), *Melipona seminigra* (n = 1) and *Tetragonisca Angustula* (n = 1) were collected in three cities of Santa Catarina State, Brazil: Santa Rosa de Lima, Rio do Sul, Iporã do Oeste and Florianópolis region characterized by the tropical climate.

Samples were dried in an oven at 30 °C for 12 h to avoid biological damages, subsequently grinded to standard the particle size and storage at −18 °C in the dark until the analysis moment.

### 2.1 Determination of moisture and ash content

The moisture (925.09) content was determined using 3 g of each geopropolis sample in porcelain caps previously dried, and then samples were placed in oven at 105 °C until constant weight [6]. Subsequently the residue of moisture content was reused to ash content (923.03) determination. The caps were heated in oven at 550 °C until constant weight [6]. Both datas were expressed in % (m/m) of moisture and % (m/m) of ash content for each geopropolis sample.

### 2.2 Extraction procedure, the yield of extraction and determination of reducing activity and the free radical scavenging potential

The details about the extraction procedure and the yield determination are available at [4]; topic 2.2.1. Briefly the extraction in two different solvents (pure methanol and pure ethanol) in a solid-liquid ratio of 3 g/10 mL were used for the determination of yield of extraction and the determination of reducing activity and the free radical scavenging potential of geopropolis samples in three different periods of extraction.

The determination of reducing activity was evaluated according to the capacity of extract to reduce the Folin-Ciocalteau reagent [8]. A hundred microliters of each geopropolis extract were added in a 10 mL glass tube with 2 mL of ultra-pure water, then 500 μL of Folin-Ciocalteau was added, and the reaction occurred after the addition of 1.5 mL of sodium carbonate (20% m/m). After 2 h, the absorbance was read in 765 nm, and the results evaluated in gallic acid equivalents (mg GAE 100^−1^g of sample) [8].

The free radical scavenging potential was determined according to the DPPH method. A methanolic DPPH solution (Abs 515nm 0.800) was added in cuvettes (2.9 mL) with 100 μL of each geopropolis sample. The absorbance was read after 30 min in the absence of light in 515 nm, and the results evaluated in ascorbic acid equivalents (mg AAE 100^−1^g of sample) and Trolox equivalent (mg TE 100^−1^g of sample) [2].

### 2.3 Polyphenolic composition by HPLC–ESI-MS/MS

For the polyphenolic determination showed in Fig. 1, Fig. 2, Fig. 3, Fig. 4, Fig. 5, Fig. 6, Fig. 7, Fig. 8, Fig. 9, Fig. 10, Fig. 11, three extraction strategies were used to investigate the free and bonded phenolic compounds. First the free phenolic compounds were analyzed using a solid-liquid extraction regarding the methodology needs [1,7].

Second, to investigate the bonded polyphenolic compounds, an acid [7] and alkaline [5] hydrolysis were used in order to release this compounds to the solution.

The chromatographic separation occurred in an HPLC-ESI-MS/MS system, coupled with mass spectrometer. The details about the extraction method and the separation conditions are available in Ref. [4].

### 2.4 Antimicrobial potential

One gram of each geopropolis sample was extracted with 5 mL of methanol. Samples were extracted in the ultrasonic bath for 30 minutes (room temperature); after that, were kept under low temperature (5 ± 2 °C) for 24 h, after that were again sonicated for more 30 minutes. The supernatant was separated in a centrifuge and reduced under low pressure until complete solvent evaporation. Subsequently, 5 mL of DMSO have used to recovery the geopropolis samples, filtered in 0.45 μm polytetrafluoroethylene syringe filter and analyzed.

Mueller Hinton agar plates with available cells of *Escherichia coli* (ATCC: 25922), *Staphylococcus aureus* (ATCC: 25923) and *Salmonella typhimurium* (ATCC: 14028) in 10^5^ CFU/mL cultivated in BHI broth were used to determinate the antimicrobial potential, according to agar diffusion method with wells technique [3].

The agar plates were perforated and 6–8 mm wells were performed. 30 μL of each geopropolis extracts (200 and 150 mg mL-1) were added in the wells followed by negative control (pure DMSO) and positive control (ciprofloxacin 0.05 mg mL^−1^). Petri plates were incubated at 37 °C for 24 h. The potential antimicrobial effect was attributed when observed halo formation surrounding geopropolis samples wells. Assays were performed in duplicate.
