# Supplementary material for: Dataset about Southern-Brazilian geopropolis: Physical and chemical perspectives
Source: Data Brief. 2020 Jan 9;29:105109. doi: 10.1016/j.dib.2020.105109 (PMC6970146; doi:10.1016/j.dib.2020.105109)
Supplement: Multimedia component 1 [file mmc1.docx]

Supplementary material 1 – Raw data of Moisture and ash content of crude geopropolis samples (related to Table 2)

| Samples | Moisture (%) | Ash content (%) |
| --- | --- | --- |
| MMS | 3.62 | 81.04 |
|  | 3.47 | 82.13 |
|  | 3.43 | 77.36 |
| MMI | 2.59 | 77,14 |
|  | 2.62 | 77,04 |
|  | 2.59 | 78,34 |
| MQS | 3.71 | 65.93 |
|  | 4.10 | 65.47 |
|  | 3.56 | 66.62 |
| MQR | 4.84 | 51.99 |
|  | 4.77 | 51.21 |
|  | 4.72 | 52.72 |
| MQF | 3.84 | 66.31 |
|  | 3.57 | 66.40 |
|  | 3.53 | 65.17 |
| MQI | 3.44 | 58,58 |
|  | 3.25 | 58,50 |
|  | 3.28 | 57,88 |
| MSS | 3.45 | 77.95 |
|  | 3.29 | 78.51 |
|  | 2.94 | 78.40 |
| MSI | 3.22 | 71,41 |
|  | 3.21 | 70,12 |
|  | 3.18 | 68,96 |
| MSeS | 8.86 | 71.90 |
|  | 8.95 | 72.03 |
|  | 8.59 | 70.52 |
| TAI | 4.12 | 2,22 |
|  | 4.02 | 2,24 |
|  | 4.24 | 2,22 |

Na: not available

Supplementary material 2 – Raw data of yield of extraction of geopropolis samples regarding pure ethanol and pure methanol as solvents over storage time (related to Table 3)

| Samples | Ethanolic extraction (mg g^-1^) | | | Methanolic extraction (mg g^-1^) | | |
| --- | --- | --- | --- | --- | --- | --- |
|  | 10 days | 20 days | 30 days | 10 days | 20 days | 30 days |
| MMS | 9.70 | 10.50 | 9.70 | 12.47 | 14.41 | 11.64 |
|  | 12.12 | 12.12 | 9.71 | 12.46 | 15.52 | 12.47 |
| MMI | 69.25 | 45.14 | 29.55 | 39.21 | 50.64 | 44.11 |
|  | 60.29 | 40.21 | 30.37 | 42.48 | 42.48 | 50.64 |
| MQS | 178.71 | 170.59 | 172.21 | 157.96 | 156.30 | 159.62 |
|  | 180.34 | 178.71 | 173.84 | 157.97 | 161.28 | 171.26 |
| MQR | 340.07 | 341.74 | 340.07 | 331.58 | 325.86 | 346.28 |
|  | 337.58 | 331.76 | 360.03 | 330.76 | 333.21 | 365.06 |
| MQF | 229.79 | 231.44 | 233.90 | 240.81 | 238.33 | 259.85 |
|  | 228.97 | 232.26 | 233.91 | 235.02 | 240.81 | 263.16 |
| MQI | 188.27 | 182.51 | 212.11 | 191.10 | 212.42 | 221.45 |
|  | 183.33 | 198.95 | 194.84 | 194.38 | 211.60 | 218.99 |
| MSS | 30.53 | 25.71 | 24.91 | 24.76 | 36.32 | 23.11 |
|  | 28.92 | 26.51 | 25.71 | 26.41 | 36.31 | 23.10 |
| MSI | 58.66 | 61.92 | 69.25 | 116.22 | 130.33 | 117.88 |
|  | 60.29 | 72.51 | 60.29 | 113.73 | 143.61 | 117.05 |
| MSeS | 23.17 | 22.11 | 23.69 | 16.61 | 24.92 | 9.97 |
|  | 23.18 | 20.53 | 22.11 | 14.95 | 25.25 | 11.63 |
| TAI | 402.16 | 397.19 | 404.64 | 198.44 | 213.32 | 197.61 |
|  | 389.75 | 399.68 | 402.16 | 194.30 | 213.33 | 206.70 |

Supplementary material 3 – Raw data of educing activity and the free radical scavenging potential of crude geopropolis samples in three different periods of extraction using ethanol as extractor agent (related to Table 4)

| Samples | Days of extraction | Ethanolic extraction | | | | | | | | |
| --- | --- | --- | --- | --- | --- | --- | --- | --- | --- | --- |
|  |  | Reducing activity (GAE mg 100g^-1^) | | | Free radical scavenging potential | | | | | |
|  |  |  |  |  | AAE mg 100g^-1^ | | | TE mg 100g^-1^ | | |
| MMS | 10 | 63.265 | 61.810 | 62.974 | 74.80 | 75.63 | 77.06 | 109.80 | 111.0221 | 113.1299 |
|  | 20 | 67.34 | 68.72 | 64.138 | 75.20 | 76.51 | 71.08 | 110.3855 | 112.315 | 104.3334 |
|  | 30 | 67.923 | 62.392 | 67.049 | 77.93 | 78.41 | 77.54 | 114.3949 | 115.1121 | 113.8261 |
| MMI | 10 | 514.966 | 459.792 | 462.696 | 560.87 | 548.53 | 540.18 | 823.12 | 805.00 | 792.73 |
|  | 20 | 491.735 | 488.831 | 494.639 | 526.52 | 522.28 | 537.56 | 772.66 | 766.42 | 788.88 |
|  | 30 | 552.717 | 535.294 | 561.429 | 594.68 | 605.03 | 608.94 | 872.79 | 888.00 | 893.74 |
| MQS | 10 | 1040.799 | 1017.563 | 1011.754 | 1160.43 | 1168.02 | 1165.25 | 1703.083 | 1714.23 | 1710.156 |
|  | 20 | 982.708 | 1061.131 | 1020.468 | 1152.35 | 1157.10 | 1139.64 | 1691.214 | 1698.19 | 1672.539 |
|  | 30 | 1055.322 | 1046.608 | 1037.895 | 1232.47 | 1206.11 | 1216.21 | 1808.909 | 1770.191 | 1785.035 |
| MQR | 10 | 1218.984 | 960.085 | 1024.810 | 250.76 | 253.96 | 257.60 | 367.96 | 372.66 | 378.01 |
|  | 20 | 1283.709 | 1230.752 | 1260.172 | 287.58 | 284.94 | 286.19 | 422.05 | 418.18 | 420.01 |
|  | 30 | 1319.013 | 1316.071 | 1324.897 | 324.02 | 325.91 | 325.51 | 475.59 | 478.36 | 477.78 |
| MQF | 10 | 1612.588 | 1700.016 | 1641.731 | 3091.98 | 3026.45 | 3054.43 | 4538.15 | 4441.88 | 4482.98 |
|  | 20 | 1729.159 | 1787.444 | 1729.159 | 3418.07 | 3364.15 | 3390.72 | 5017.20 | 4937.99 | 4977.03 |
|  | 30 | 1933.157 | 2370.296 | 1904.014 | 3618.94 | 3589.07 | 3632.37 | 5312.30 | 5268.43 | 5332.03 |
| MQI | 10 | 1566.956 | 1596.045 | 1502.959 | 1607.44 | 1652.70 | 1612.77 | 2357.22 | 2423.72 | 2365.05 |
|  | 20 | 1532.049 | 1514.595 | 1479.688 | 1681.88 | 1646.61 | 1667.88 | 2466.58 | 2414.77 | 2446.01 |
|  | 30 | 1508.777 | 1572.774 | 1590.227 | 1955.09 | 1961.11 | 1947.29 | 2867.95 | 2876.79 | 2856.50 |
| MSS | 10 | 427.294 | 441.994 | 447.874 | 973.27 | 984.86 | 973.95 | 1428.111 | 1445.139 | 1429.11 |
|  | 20 | 439.054 | 483.155 | 447.874 | 1038.93 | 1017.18 | 999.34 | 1524.572 | 1492.607 | 1466.411 |
|  | 30 | 486.095 | 427.294 | 468.455 | 1115.17 | 1107.03 | 1112.08 | 1636.573 | 1624.613 | 1632.028 |
| MSI | 10 | 1633.580 | 1679.704 | 1645.111 | 1917.35 | 1894.86 | 1970.05 | 2812.55 | 2779.50 | 2889.97 |
|  | 20 | 1376.058 | 1410.651 | 1399.120 | 1790.04 | 1775.35 | 1775.35 | 2625.52 | 2603.93 | 2603.93 |
|  | 30 | 1295.340 | 1295.340 | 1387.589 | 2071.71 | 2052.54 | 2073.05 | 3039.32 | 3011.15 | 3041.28 |
| MSeS | 10 | 69.267236 | 68.11439 | 66.0969 | 62.65 | 61.71 | 64.78 | 91.95009 | 90.56731 | 95.08982 |
|  | 20 | 67.249752 | 68.4026 | 67.53796 | 65.69 | 65.29 | 63.88 | 96.42649 | 95.83889 | 93.75954 |
|  | 30 | 65.808693 | 73.87863 | 73.59041 | 72.22 | 70.64 | 68.82 | 106.0146 | 103.699 | 101.0126 |
| TAI | 10 | 1337.085 | 1178.978 | 1389.787 | 351.41 | 355.16 | 336.55 | 515.83 | 521.34 | 494.00 |
|  | 20 | 1178.978 | 1354.652 | 1161.410 | 304.51 | 312.35 | 329.46 | 446.93 | 458.45 | 483.58 |
|  | 30 | 1495.192 | 1378.076 | 1237.536 | 354.85 | 326.12 | 366.13 | 520.88 | 478.67 | 537.45 |
